# The first complete mitochondrial genome from the family Solasteridae, *Crossaster papposus* (Echinodermata, Asteroidea)

**DOI:** 10.1080/23802359.2020.1846001

**Published:** 2021-01-05

**Authors:** Sang-Eun Nam, Sung Ah Kim, Tae-Yoon S. Park, Jae-Sung Rhee

**Affiliations:** a Department of Marine Science, College of Natural Sciences, Incheon National University, Incheon, South Korea; b Division of Polar Earth-System Sciences, Korea Polar Research Institute, Incheon, South Korea; c Polar Science Department, University of Science & Technology, Daejeon, South Korea; d Research Institute of Basic Sciences, Incheon National University, Incheon, South Korea

**Keywords:** Mitochondrion, sunstar, Valvatida, Solasteridae, phylogeny

## Abstract

The common sunstar, *Crossaster papposus*, belongs to the family Solasteridae whose ordinal classification has been unstable. Here, for the first time, we assembled and annotated the complete mitochondrial genome of the common sunstar, *C. papposus* Linnaeus, 1767. The circular genome of *C. papposus* is 16,335 bp in length and contains 13 protein-coding genes (PCGs), 22 transfer RNA (tRNA) genes, a control region, and large and small ribosomal subunits. The overall genomic structure and gene arrangement were identical to the reported mitochondrial genomes of sea star species, and a phylogenetic analysis of 13 PCGs recovers a closest relationship with the derived cluster of the paraphyletic order Valvatida.

The common sunstar, *Crossaster papposus* (Linnaeus 1767) is a conspicuous and ubiquitous starfish in the North Atlantic. *C. papposus* has a wide circumboreal distribution in all northern seas from the coastal region to oceanic depths (Clark and Downey 1992). The genus *Crossaster* belongs to the large family Solasteridae. Since Blake (1987) reinstated the order Velatida, the Solasteridae has long been regarded as a member of the order Velatida. However, based on the valvatacean phylogeny acquired using three genes (12S, 16S, and early-stage histone H3), Mah and Foltz (2011) assigned the Solasteridae to the order Valvatida. In the recent phylogenetic analysis of seven asteroid orders (Linchangco et al. 2017), the Valvatida appeared to be paraphyletic, and the members of the Solasteridae were not included in the tree. Here we present the complete mitogenome of *C. papposus*, which will be the first case within the Solasteridae. The whole mitogenome of *Crossaster* species will be useful to understand the phylogenetic context of the Solasteridae within the Asteroidea, as well as their diversity, taxonomy, and geographic distribution.

A specimen of *C. papposus* was collected in 2017 from the Beaufort Sea (82°46′1.7′′N, 42°32′52.4′′W) using a remotely operated underwater vehicle (ROV) of Monterey Bay Aquarium Research Institute (MBARI). The voucher specimen was registered both in the Research Institute of Basic Sciences of Incheon National University and in the Korea Polar Research Institute (Species ID: Echinodermata-01; Specimen ID: KOPRI-Benthos-01). Mitochondrial genomes were recovered by *de novo* assembling from Illumina shotgun sequence data. Genomic DNA was isolated using a QIAamp DNA Blood Mini kit (Qiagen, Hilden, Germany). Based on the manufacturer’s instructions (Illumina, San Diego, CA), a genomic library was constructed using a TruSeq Nano DNA Kit by Macrogen, Inc. (Seoul, South Korea). Raw reads were obtained from the sample that passed quality control by Illumina HiSeq platform. After the trimming process on the raw reads, *de novo* assembly was performed using SPAdes version 3.11.1 (Bankevich et al. 2012). Genomic features and annotations were performed using MITOS2 (Bernt et al. 2013) and tRNAscan-SE 2.0 (Lowe and Eddy 1997). The annotated gene structure was further confirmed using NCBI-BLAST (http://blast.ncbi.nlm.nih.gov). Mitochondrial genome of *C. papposus* was aligned with mitogenomes from other echinoderm genera, as well as two outgroup taxa from the genus *Balanoglossus*, and 13 PCGs were extracted for phylogenetic analysis (Nam et al. 2020). jModelTest version 2.1.10 (Darriba et al. 2012) was used to determine the best substitution model with an appropriate partitioning scheme, and a maximum likelihood phylogenetic analysis was conducted with 1000 bootstrap replicates in the PhyML version 2.4.5 (Guindon and Gascuel 2003).

The complete mitochondrial genome for *C. papposus* contained 13 PCGs, 22 tRNA genes, 2 rRNA genes, and a control region. The mitogenome for *C. papposus* (GenBank accession no. MW046047) was 16,335 bp long and had a GC content of 32.8% with an AT bias (A: 35.5%; T: 31.7%; G: 12.7%; C: 20.1%). The arrangement of genes and gene composition were identical to those of other starfishes. Our *COI* sequence was identical to the partial *COI* sequence (841 bp; GenBank accession no. MK270384) of *C. papposus* collected from the Baffin Bay (Ringvold and Moum 2020).

Although data for the order Velatida is lacking, our phylogenetic analysis using mitogenome data shows a similar ordinal relationship within the Asteroidea to the result of Linchangco et al. (2017). Our result resolved a close relationship of *C. papposus* to the derived cluster of the paraphyletic Valvatida, together which form a sister group to the order Spinulosida (Figure 1). This is in line with the previous suggestion that the Solasteridae belongs to the order Valvatida (Mah and Foltz 2011). Although different taxa of the Valvatida were used for phylogenic analysis, paraphyly of the order Valvatida was produced both in our study and in Linchangco et al. (2017). Future researches on the paraphyly of the Valvatida and the mitogenome data from the order Velatida are required.

**Figure 1.**
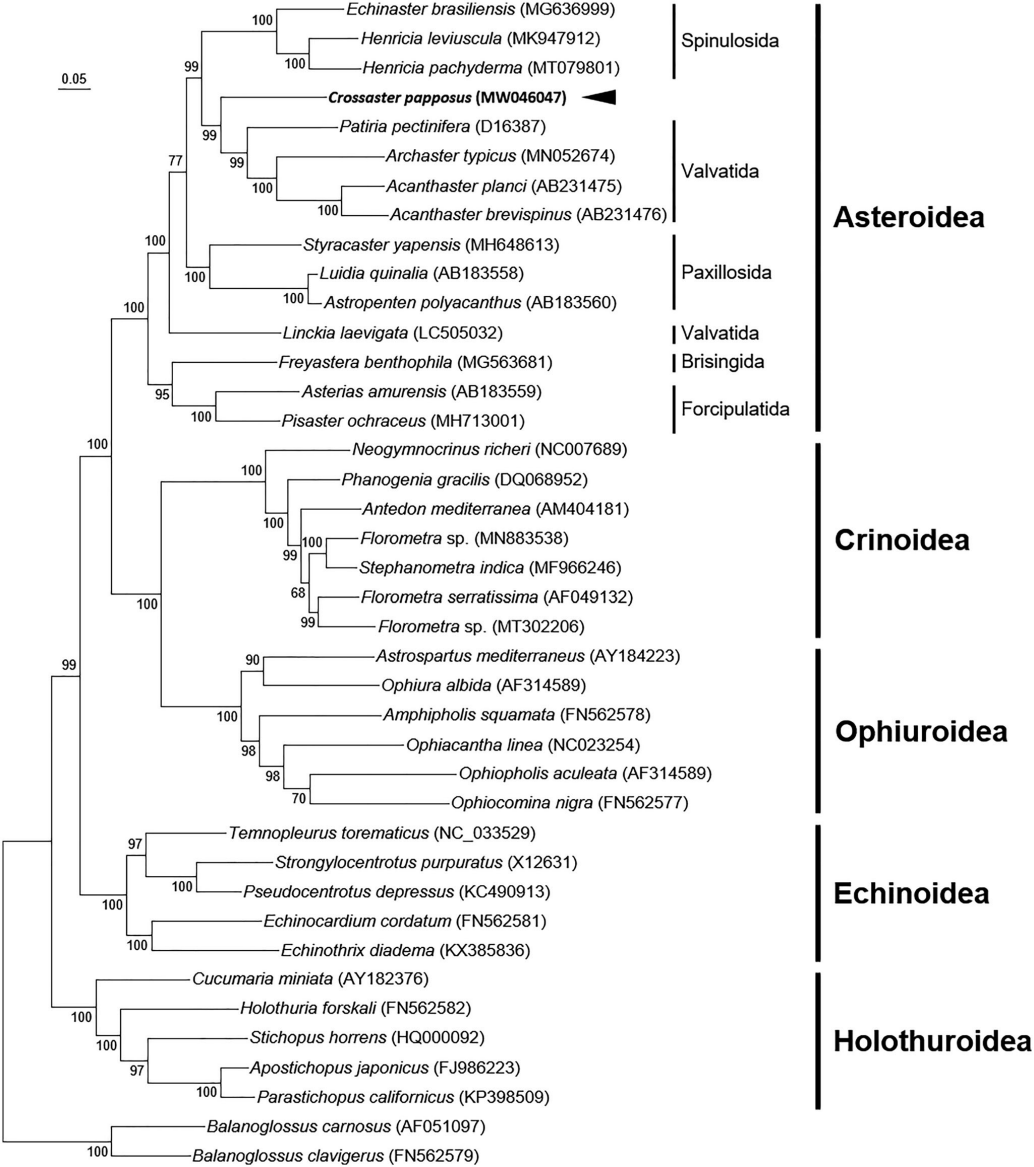
Maximum-likelihood (ML) phylogeny of 38 echinoderms (15 asteroids including *C. papposus*, 7 crinoids, 5 echinoids, 5 holothuroids, and 6 ophiuroids) and two *Balanoglossus* mitogenomes as an outgroup based on the concatenated nucleotide sequences of entire protein-coding genes (PCGs). Numbers at nodes represent ML bootstrap percentages (1000 replicates). DDBJ/EMBL/Genbank accession numbers for published sequences are incorporated. The black arrow indicates the *C. papposus* analyzed in this study.

## Data Availability

The data that support the findings of this study are openly available in the National Center for Biotechnology Information (NCBI) at https://www.ncbi.nlm.nih.gov, accession number MW046047.
